# Impaired Glucose Tolerance in a Mouse Model of Sidt2 Deficiency

**DOI:** 10.1371/journal.pone.0066139

**Published:** 2013-06-11

**Authors:** Jialin Gao, Xuefan Gu, Don J. Mahuran, Zhugang Wang, Huiwen Zhang

**Affiliations:** 1 Department of Pediatric Endocrinology and Genetic Metabolism, Xinhua Hospital, Shanghai Institute for Pediatric Research, Shanghai Jiao Tong University School of Medicine, Shanghai, China; 2 Department of Laboratory Medicine & Pathobiology, Research Institute, The Hospital for Sick Children, University of Toronto, Toronto, Canada; 3 Shanghai Research Centre for Model Organisms, Shanghai, China; Consiglio Nazionale delle Ricerche, Italy

## Abstract

Sidt2 was identified as a novel integral lysosomal membrane protein recently. We generated global Sidt2 knockout mice by gene targeting. These mice have a comparatively higher random and fasting glucose concentration. Intraperitoneal and oral glucose tolerance tests in Sidt2 knockout mice indicated glucose intolerance and decreased serum insulin level. Notably, the Sidt2^−/−^ mice had hypertrophic islets compared with control mice. By Western blot and immunofluorescence, Sidt2^−/−^ mouse islets were shown to have increased insulin protein, which actually contained more insulin secretory granules than their controls, demonstrated by electromicroscopy. Consistent with the in vivo study, isolated islet culture from the Sidt2^−/−^ mice produced less insulin when stimulated by a high concentration of glucose or a depolarizing concentration of KCl. Under electromicroscope less empty vesicles and more mature ones in Sidt2^−/−^ mice islets were observed, supporting impaired insulin secretory granule release. In conclusion, Sidt2 may play a critical role in the regulation of mouse insulin secretory granule secretion.

## Introduction

The lysosomal membrane has long been seen as a physical barrier that allows the acidification of the lumen in order to promote the turnover of both extra- and intra-cellular macromolecules. In recent years this membrane has been found to play more varied roles in the functions of the lysosomes. For example, the negatively charged lipids that are abundant in the lysosomal membrane are needed to present some substrates to hydrolytic enzymes [1]. Receptors on the lysosomal membrane are needed to transport the degradation products out of the lysosome for recycling [2]. And some proteins on the lysosomal membrane are needed to promote specific interaction and fusion events with various cellular organelles, including autophagosomes, endosomes and plasma membrane [3]. The lysosomal membrane also is directly involved in micro-, macro-, and chaperone mediated autophagy[4]. Abnormalities in these pathways have been linked with several neurodegenerative diseases [5], [6].

In the past 30 years, lysosomes have also been subjected to investigation on association with insulin secretion, and human diabetes, as well as in animal models of diabetes. As early as to 1979, plasma lysosomal enzyme N-acetyl-beta, D-glucosaminidase activity has been found to increase in human diabetes[7]. Subsequently, certain more lsosomal glycosidases were found elevated in human type 1 diabetes [8]. In 1983, Dr. Lundquist reported that acid amyloglucosidase activity was important for glucose-stimulated insulin secretion[9]. Later, more data supported that the lysosomal system participate the secretory process of the insulin from the pancreatic beta-cell[10]. In a nonobese animal model of noninsulin-dependent diabetes mellitus, the Goto-Kakizaki (GK) rat, the lysosomal enzyme activities in islet tissue showed an abnormal pattern, and this dysfunction was presumed to convey impairment of glucose-induced insulin release[11]. Recent studies provide evidence that basal autophagy mediated by lysosome is necessary to maintain the architecture and function of pancreatic beta cells and its induction in diabetic mice protects beta cell against damage by oxidative stress[12].

Type 2 diabetes is a complex metabolic disorder predominantly characterized by defects in insulin secretion in early phase. Insulin secretary vesicles shared some similarities with lysosomes. First, both their membrane concentrate lysosome associated membrane proteins (LAMPs) and vesicle associated membrane protein (VAMPs)[13]; Secondly, insulin release after secretary dense-core vesicle fuse with the plasma membrane was similar to lysosome exocytosis [14]. Some novel lysosomal membrane protein may play a vital role in diabetes.

Currently, approximately 50 integral and peripheral membrane proteins associated with the lysosome have been identified [15], [16]. Discovery of such proteins and elucidating their functions is important for understanding the dynamics of lysosomes. In a recent proteomic study of lysosomal proteins [15], [17], we identified SID1 transmembrane family, member 2 (Sidt2) as a novel lysosomal membrane protein candidate. The Sidt2 gene encodes an 832-amino acid protein with a calculated molecular mass of 94.5 kDa. Further studies confirmed that Sidt2 is a lysosomal integral membrane protein,and is widely expressed in tissue [18].

Sidt2 was conserved in human, chimpanzee, Rhesus monkey, dog, cow, rat, mouse, chicken, and C.elegans. By gene ontology, Sidt2 was presumed to be an RNA membrane transporter, like another SID1 transmembrane family protein, Sidt1[19]. Overexpression the ScSidT2 protein in fathead minnow epithelial cells could increase the uptake of exogenous dsRNA [20]. But the true cellular role of Sidt2 has not been examined in the whole animal level.

Generating a knock out mouse model by targeting the specific gene of interest is commonly used to help determine the function of the gene's protein product in vivo. Using such models several lysosome proteins have been elucidated, such as LAMP1[21] and LAMP2[22]. In this study, we generated Sidt2 conditional knockout mice model, termed as Sidt2^Flox/Flox^ (Sidt2^F/F^) mice. Using Cre-LoxP system [23], we have obtained the Sidt2-deficient (Sidt2^−/−^) mouse. From our evaluation of these mice we demonstrated that Sidt2 may play a vital role in insulin granule secretion.

## Materials and Methods

This study was approved by the Institutional Review Ethics Board of Xinhua Hospital, which received agreement SYXK (Hu) 2008-0052 of mouse laboratory from Science and Technology Commission of Shanghai Municipality. All mice were housed in animal laboratory center with standard temperature and humidity under the guideline for the welfare and use of animals in Xinhua Hospital. Every effort was made to minimize mouse suffering during the manipulations.

### Generation of Global Sidt2 KO Mouse

**Figure 1:**
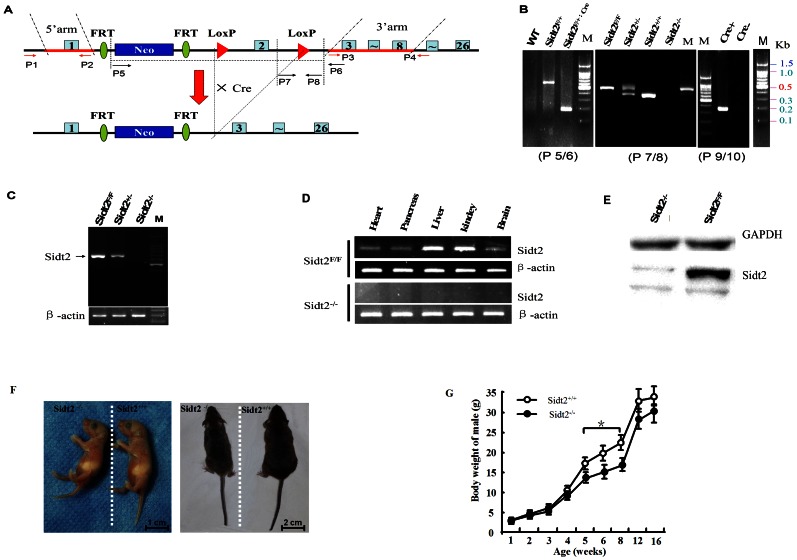
Targeting strategy and generation of Sidt2 conditional knockout mice. (A) Schematic of the gene targeting strategy. The neomycin resistance cassette and flanked LoxP1 sequences were inserted into intron 1 for positive selection. The second LoxP2 sequences were inserted into intron 2. (B) The results of PCR genotyping. Primers 5 and 6 were used for the genotyping of floxed mice Sidt2^F/+^, Sidt2^F/+: Cre^ (Sidt2^+/−^) and WT (Sidt2^+/+^). Primers 7 and 8 were used to differentiate Sidt2^−/+^, Sidt2^−/−^, Sidt2^+/+^ and Sidt2^F/F^, while the primers 9 and 10 were used to confirm Cre deletion. (C) RT-PCR analysis of Sidt2 mRNA (extracted from tail tissue). (D) Sidt2 mRNA detection in various tissues with β-actin loaded as an internal control. (E) Western blot analysis of Sidt2 protein in the liver of KO mice with GAPDH loaded as an internal control. (F) Appearance of mice at birth and as adults. (G) Body weight change of Sidt2^−/−^ mice (n = 35–50).

The Sidt2 was targeted by insertion of the LoxP site tagged Neo-resistance gene cassette in intron 1 and a LoxP site in intron 2. Thus, the second exon of the gene was flanked with LoxP sites and could be deleted by conditionally expressed Cre recombinase. Targeting vector DNA was electroporated into 129S_V_/E_V_ mouse embryonic stem cells (SCR012). Targeting vector design is shown in Fig.1A. Positive clones were selected with G418 and GanC, and verified by PCR with two pairs of primer (P1P2 and P3P4, sequence provision if required) and sequencing. Recombinant ES cells were injected into C57BL/6J mouse blastocysts to produce chimeras. The chimeras were crossed with C57BL/6J mice to produce heterozygous Sidt2-floxed offspring. Homozygotes were obtained by intercrossing heterozygotes.

To delete Sidt2, Sidt2^F/F^ mice were crossed with EII-Cre transgenic mice that express Cre in whole body [24], generating EII-Cre^+/−^:Sidt2^F/+^ mice, hereafter referred to as heterozygote KO mice (Sidt2^−/+^). Intercrossing with heterozygote Sidt2^−/+^ mice, homozygous mice (Sidt2^−/−^) were obtained. The deletion of the LoxP-flanked fragment (Flox) and the Cre site was confirmed by PCR and validated by sequencing using three pairs of primer (P5P6, P7P8, and P9P10, sequences provision if required).

### Assessment of Glucose Metabolism

Intraperitoneal glucose tolerance tests (IPGTTs) were performed after a 12-h fasting in Sidt2−/− and Flox mice. Blood was sampled from the tail vein before and 30, 60, and 120 min after intraperitoneal injection of 1.5 g/kg glucose. Oral glucose tolerance tests (OGTTs) were also performed after overnight (at least 12 h) fast. Each mouse was given an oral glucose load 2 g/kg body weight. Blood samples were collected from the tail vein at time 0 (prior to the glucose loading), 30, 60 and 120 minutes after glucose loading for blood glucose. The time point of 15 minutes after glucose loading was added for insulin determination. Blood glucose concentrations were measured by blood glucose meter (Johnson & Johnson). Insulin was analyzed by Insulin ELISA Kit (Millipore). Insulin tolerance test (ITT) was carried by insulin injection intraperitoneally at 0.05 units/kg and observation of tail blood glucose variation.

### Islet Isolation and Insulin Release

To obtain pancreatic islets, pancreas were removed and the islets isolated by collagenase digestion, using a previously published protocol [25]. The islets were individually dissected under a stereomicroscope. Batches of 10 similarly sized islets were collected and incubated in RPMI 1640–10% fetal calf serum at 37°C in 5% CO2 for 2 h. These islets were washed and preincubated in 0.5% (w/v) bovine serum albumin–Krebs-Ringer HEPES-buffered saline in 2.8 mM glucose at 37°C in 5% CO_2_ for 30 min and then transferred to 0.5% (w/v) bovine serum albumin–Krebs-Ringer HEPES-buffered saline in 2.8 mM glucose, stimulatory 20 mM glucose alone, or 30 mM KCl with 2.8 mM glucose. After incubation at 37°C in 5% CO_2_ for 30 min, the supernatants were collected for insulin determination as previously described [26]. Insulin level was assayed with above mentioned Insulin ELISA Kit.

### Transmission Electron Microscopy (TEM)

Pancreatic islets were fixed in 2.5% glutaraldehyde for 1 h, treated with 1% osmium tetroxide, dehydrated and embedded in Durcupan (Sigma-Aldrich). Samples were then sectioned (60 nm), mounted on Cu-grids and contrasted with uranyl acetate and lead citrate and examined by electron microscopy (EM-1200EX, JEOL).

### RNA and Protein Analysis

Expression of the Sidt2 gene was investigated by reverse transcriptase (RT)-PCR method as previously reported(sense 5′-ATGTGGTGGTGGTAGTGAAG -3′, antisense 5′-AGATACACCACCACCATCAC -3′, and the annealing temperature 56°C)[18]. Insulin mRNA was analyzed by the RT-PCR (Ins-1: sense 5′CCACCTGGAGACCTTAATGGG3′, antisense 5′TGCTACGGATGGACTGTTTGT3′; Ins-2: sense 5′AGCCTATCTTCCAGGTTATTGTTTC3′, antisense 5′GGTAGTGGTGGGTCTAGTTGCA 3′). For analysis of Glucagon, the primer (sense 5′CAGCGACTACAGCAAATACC3′, antisense 5′TCCCTGGTGGCAAGATTA3′) were used. Tissues were homogenized in lysis buffer (Beyotime). Homogenates were centrifuged at 12,000× g at 4°C for 10–20 min. The lysate was analyzed by Western blot using the anti-insulin (Abcam, ab14042) or the anti-Sidt2 (Abcam) antibody. Western blot analysis was performed as described previously. The gray values were analyzed all by BandScan 5.0, and the error bars indicated the statistical differences in three independent experiments.

### Immunostaining

Paraffin-embedded pancreas from Sidt2^−/−^ and control mice were deparaffinized. Sections were treated in an Antigen Unmasker (Pickcell) and stained with anti insulin (1∶500) and anti glucagon (1∶300) antibodies (Abcam), and revealed using Alexa dye-conjugated secondary antibodies (Invitrogen) for multiple labeling. Sections were mounted in Vectashield with DAPI (Beyotime), and images were captured and analyzed using a Zeiss AxioSkop2 microscope.

### The β cell Counting and Islet Morphological Examination

To quantities the beta cells, the beta cell area marked by positive insulin staining was divided by the number of nuclei within this area. We counted 4 islets in 8 sections per mouse model. Five Sidt2−/− mice and six controls were used respectively. The islets size measure was conducted according to pervious report[27].

### Statistical Analyses

Results are expressed as means ± standard errors of means (SEM). Differences between experimental groups were analyzed by unpaired Student t-test. Significance of differences was set at p<0.05.

## Results

### Generation of Sidt2 Conditional Knockout and Sidt2-deficient Mice

Sidt2 gene is encoded by 26 exons that have two transcripts both of which contain exon 2. Thus, the targeting vector is designed to conditionally disrupt exon 2 by the Cre-loxP system (Figure 1A). Mice homozygous for the Sidt2^Flox^ allele (Sidt2^F/F^ mice), which were expected to express intact Sidt2, were born healthy and fertile without any noticeable pathological phenotypes (Figure 1F). RT-PCR revealed the presence of Sidt2 expression in Sidt2^F/F^ mice (Figure 1C, D), suggesting that Sidt2 is efficiently expressed from the Sidt2^F/F^ allele.

Then homozygous Sidt2^−/−^ (KO) mice were obtained when heterozygous Sidt2^+/−^ mice were intercrossed. The results of PCR genotyping are shown in Figure 1B. No Sidt2 mRNA was detected in the tail of homozygous Sidt2^−/−^ mice with primers covering Sidt2 exon 2 as expected (Figure 1C). The Sidt2 transcript was found in liver, kidney, brain, heart, and pancreas, of Sidt2^F/F^ mice, but not in tissues of the KO mice (Figure 1D). The Western blot also showed that Sidt2 protein was apparently decreased in the KO mice (Figure 1E). The Sidt2^−/−^ allele intercross progeny demonstrated a statistically significant departure from Mendel's law (unreported data), but there was no significant differences in their weights or appearance as compared to wild-type controls as newborns (homonymous as Sidt2^+/+^, WT or control) (Figure 1F). However, at ≥5 weeks of age, the male Sidt2^−/−^ mouse weighed significantly less than controls (Figure 1G), with some animals appearing grossly smaller in size (Figure 1F). Interestingly, in female Sidt2^−/−^ mice, there was no significant difference in appearance or body weight, as compared to their wild-type controls (unreported data).

### Altered Glucose Metabolism in Sidt2^−/−^ Mouse

**Figure 2:**
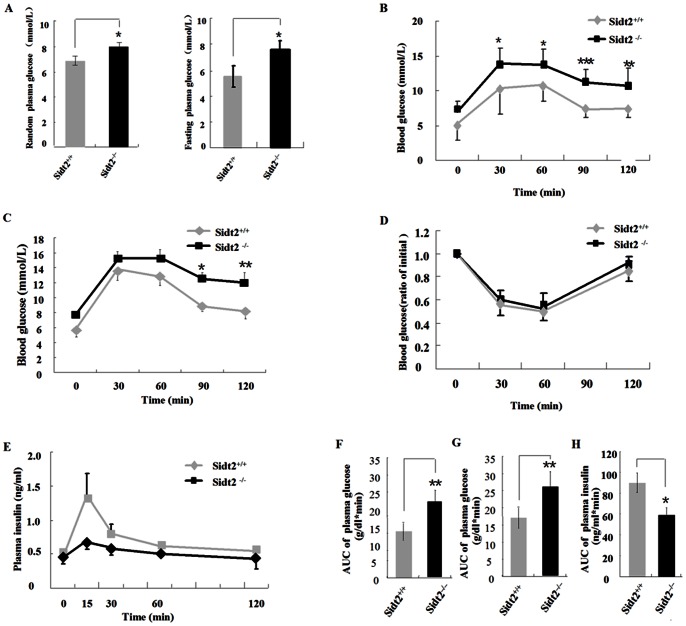
Glucose metabolism is dysfunctional in Sidt2^−/−^ mice. (A) Random and fasting blood glucose at 6 months of age. (B) Oral glucose tolerance tests at 6 months of age (n = 12). (C) Intraperitoneal glucose tolerance tests at 6 months of age (n = 12). (D) Insulin tolerance tests (n = 10). (E) Plasma insulin levels after glucose stimulation intraperitoneally at 1.5 g/kg body weight (n = 12). (F, G and H) AUC_0–120 min_ of blood glucose and plasma insulin shown in pane B, C and E, respectively. All values are the means ± SEM. * P<0.05, ** P<0.01, *** P<0.001.

Sidt2^−/−^ mice showed significant differences in their plasma glucose and insulin levels as compared to Sidt2^+/+^ mice (in the following experiment, Sidt2^+/+^ mice were used as controls instead of the Sidt2^F/F^ mice). Both random and fasting plasma glucose levels in the Sidt2^−/−^ male mice were significantly increased over those of male Sidt2^+/+^ mice at six months of age (Figure 2A). Both oral and intraperitoneal glucose tolerance tests (OGTT and IPGTT) demonstrated higher blood glucose levels in Sidt2^−/−^ mice. OGTT showed significant differences in 90 and 120 minute time points (Figure 2B), while in IPGTT, the difference was apparent at 30–120 minutes after intraperitoneal glucose loading (Figure 2C). The area under the curve (AUC)_0–120 min_ of blood glucose was also significantly higher in Sidt2^−/−^ mice than that in controls (Figure 2F). In parallel with impaired glucose tolerance, insulin responses to glucose at the early phase were lower in Sidt2^−/−^ mice (Figure 2E) and the AUC_0–120 min_ of their plasma insulin significantly decreased as compared to controls (Figure 2G). Whole-body insulin sensitivity was assessed by the insulin tolerance test (ITT), and no apparent differences were observed between Sidt2^−/−^ and control mice (Figure 2D).

### The Age Related Glucose Intolerance Changes in Sidt2^−/−^ Mouse

**Figure 3:**
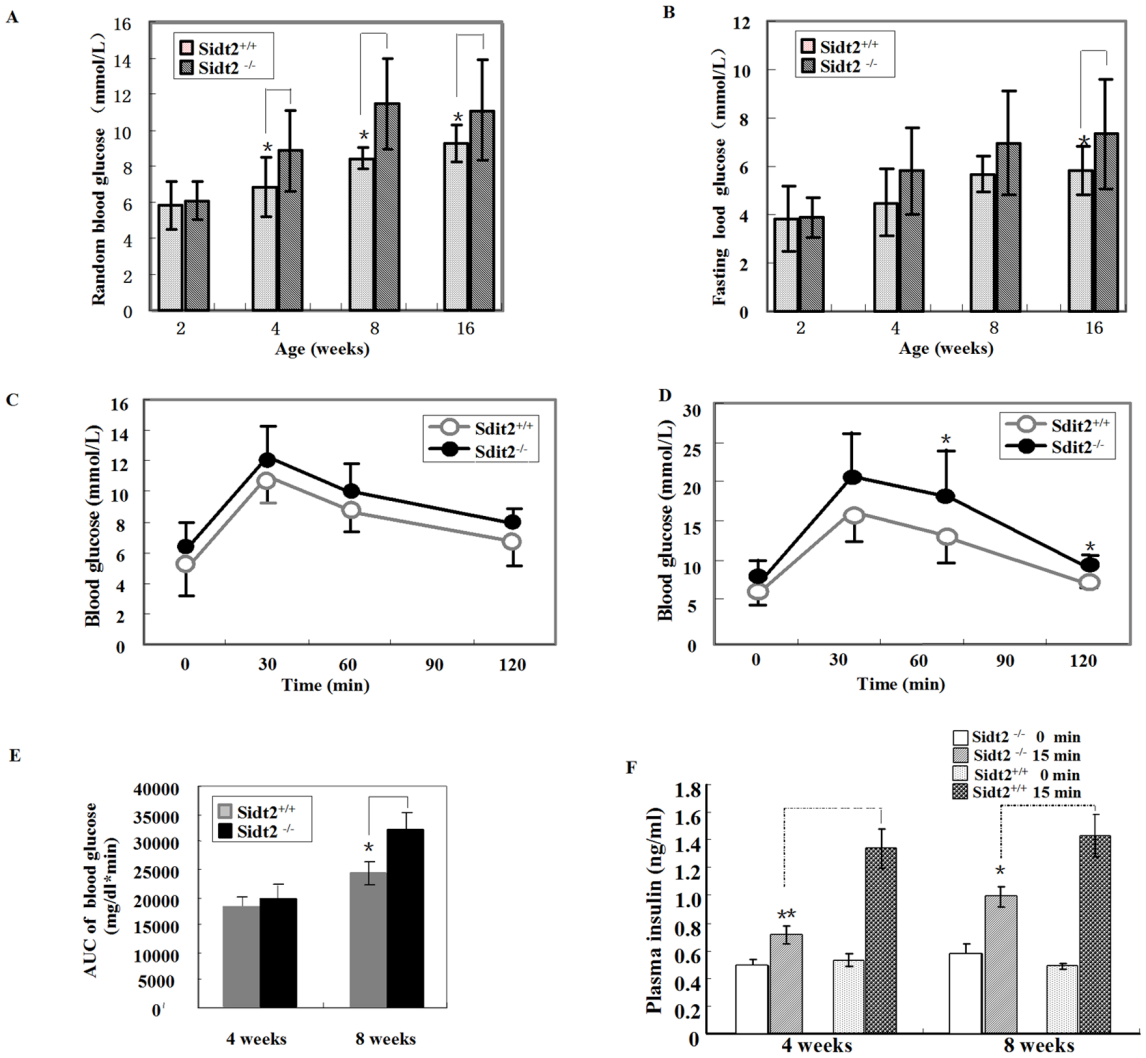
Blood glucose changes in Sidt2^−/−^ mice at different ages. (A) Random blood glucose levels in 2–16 weeks of age (n = 10–12). (B) Fasting blood glucose levels in the two groups at different age (n = 10–15, fasting overnight). (C) IPGTT at 4 weeks old (n = 8). (D) IPGTT at 8 weeks of age (n = 12). (E)AUC_0–120 min_ of the blood glucose shown in panel C and D. (F) Plasma insulin level after glucose stimulation intraperitoneally at 1.5 g/kg body weight. Data is presented as mean ± SEM (n = 7–9). All values are the means ± SEM. * P<0.05, ** P<0.01.

Assessment of random blood glucose levels at different age stages showed that Sidt2^−/−^ mice exhibited significantly higher glucose levels than controls as early as 4 weeks of age (Figure 3A). The 12-h-fasted blood glucose levels also exhibited increases in Sidt2^−/−^ mice, which became significant after 16 weeks of age (Figure 3B). Earlier as at 4 weeks of age, the IPGTT produced higher glucose levels in Sidt2^−/−^ mice although without significance than controls (Figure 3C). AUC_0–120 min_ of blood glucose was also increased and paralleled the glucose level (Figure 3E). At 8 weeks of age, AUC _0–120 min_ of blood glucose was significantly higher than that in control mice (Figure 3D, E). The serum insulin response levels at 15 and 30 min after i.p glucose injection are a reflection of the secretory function of islet β-cells. By assessing the primary secretion phase at different weeks of age in mice, we found that the Sidt2^−/−^ mice have abnormal insulin secretion both in the nonage stages (4 weeks) and adult period (after 8 weeks) (Figure 3F). At the 15 minute time point after glucose challenge, serum insulin levels of Sidt2^−/−^ mice were only 1–1.5 fold of their fasting levels, whereas Sidt2^+/+^ mice had levels ∼3-fold greater than those observed during fasting (Figure 3F).

### Increased Insulin Expression in Sidt2^−/−^ Mice

**Figure 4:**
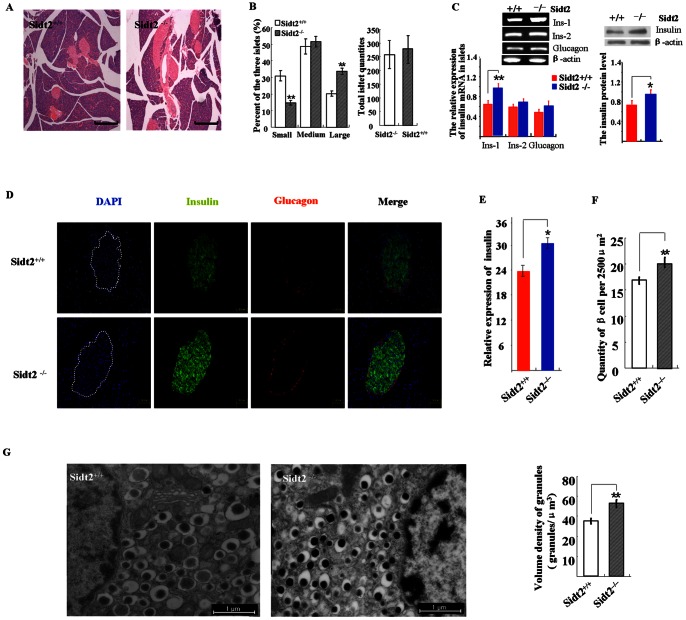
Increased insulin protein level in Sidt2^−/−^ mice. (A) H&E staining of islets in pancreatic sections. Bars: 1 mm. (B) The percentage of three islet types. Quantity of 636 islets from three Sidt2 −/− mice and 872 islets from four Sidt2 +/+ mice were determined. Total islets quantities is presented as mean ± SEM. ** P<0.01. (C) mRNA (left) and Western blot (right) analysis of insulin expression in the islets. The glucagon mRNA was also analyzed (left). * P<0.05, n = 12. (D) Immunofluorescence staining for insulin (green), glucagon (red) and DAPI (blue) in pancreatic sections. (E) Histogram showing the relative insulin expression levels calculated by the fluorescence signals in Panel D. * P<0.05, n = 4–6. (F) Count of beta cells. (G) TEM images of β-cells.

Compared to controls, Sidt2^−/−^ islets often exhibited hypertrophia volume (Figure 4A). We divided the islets into three groups: small islets (0–5000 um^2^), medium islets (5000–10000 um^2^), and large islets(>10000 um^2^). In Sidt2^−/−^ pancreas, the number of small islets was significantly lower than that in Sidt2^+/+^, and the large islets number was significantly greater (Figure 4B). However, there was no significant difference in total islet number between the two groups (Figure 4B). Islets were analyzed for their insulin mRNA and protein levels by RT-PCR and Western blotting, which indicated an increased insulin protein levels in Sidt2^−/−^ mice compared to control mice (Figure 4C). However, there is no significant difference for glucagons mRNA levels between the two groups. This increased insulin expression was also confirmed by immunostaining for insulin on pancreatic sections (Figure 4D, E). The number of β-cells was also significantly increased in islets of Sidt2^−/−^ islets (Figure 4F). Notably, by TEM analysis, we found that the β-cells of Sidt2^−/−^ islets contained more insulin secretory granules (Figure 4G), which was consistent with the increased insulin expression level observed by Western blot analysis (Figure 4C).

### Loss of Sidt2 Decreased Insulin Secretory Granules Release

**Figure 5:**
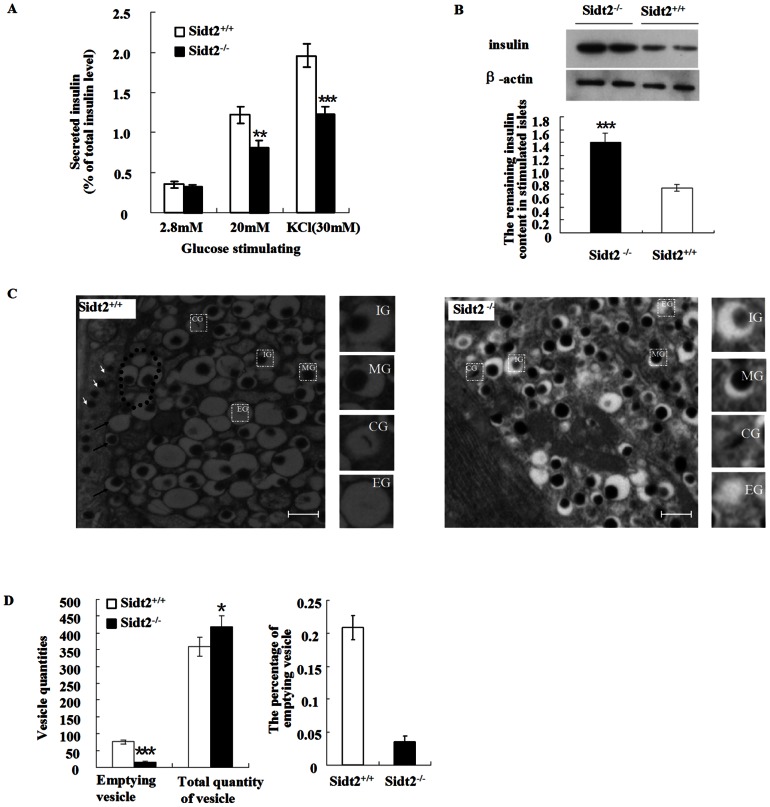
Loss of Sidt2 decreased insulin granule exocytosis. (A) GSIS assay of islets isolated from two groups. Islets incubated in 2.8 mM glucose were stimulated with 20 mM glucose and then 30 mM KCl. The total insulin levels were the sum of the released insulin and the remaining islet insulin content after stimulation. Values represent means ± SEM. * P<0.01; *** P<0.001 (n = 6). (B) The insulin protein remaining in the islet post stimulation (means ± SEM, at least three independent experiments). (C) TEM showing the secretory granules in islet β-cells. Secretory vesicle tethering and docking (dotted line) as well as fusion with plasma membrane (black arrows) and releasing of insulin granules (white arrows) were seen in control islets, while rarely seen in Sidt2−/− islets. The small boxes were the four subtypes of insulin granules; IG, immature granules, MG, mature granules, CG, crystal containing granules, EG, empty granules. Bars: 0.5 µm. (D) Quantities of empty secretory vesicles.

Sidt2 deficiency suppressed high glucose (20 mM) and KCl-stimulated insulin release but did not affect basal insulin release in the presence of 2.8 mM glucose (Figure 5A). However, total insulin in Sidt2−/− islets post stimulation was not reduced. On the contrary, Sidt2−/− islets exhibited increased insulin content as compared to control islets (Figure 5B). Electron micrographs showed that the number of empty secretory vesicles was decreased, whereas insulin granules were increased in Sidt2^−/−^ islets compared to controls (Figure 5C, D). To release their insulin contents, docking and fusion of secretory granules with the plasma membrane is necessary. In Sidt2^+/+^ islets, electron micrographs showed secretory vesicle tethering and docking with plasma membrane, as well as fusion and releasing of the insulin (Figure 5C).

## Discussion

To characterize the pathophysiological roles of Sidt2, a Sidt2 conditional knockout mouse model (Sidt2^F/+^, Sidt2^F/F^)was created. In this study, we obtained a tissue-unspecific Sidt2 KO model by intercrossing it with EII-Cre mice. There were no obvious differences in appearances between WT and Sidt2 KO mice at birth. However, Sidt2^−/−^ mice show reduced weight and smaller size at growth phase, which stimulated the glucose determination in this study.

Sidt2 deficiency resulted in glucose metabolic dysfunction, which manifested as increased random blood glucose level and impaired glucose tolerance. Adult Sidt2^−/−^ male mice have a decreased glucose tolerance. Their plasma levels of insulin were not increased as was the normal control group, in phase of after glucose injection, suggesting impairment of the islet function in Sidt2 deficiency mice. Peripheral insulin resistance has been reported to adversely affect insulin secretion, ultimately resulting in pancreatic exhaustion. So, we determined in vivo insulin sensitivity by an i.p. ITT in Sidt2^−/−^ mice. However, there was no apparent insulin resistance in Sidt2 deficient mice. Pancreatic islets of Sidt2^−/−^ mice are morphologically abnormal, e.g. hypertrophic volumes and increased quantities of large size islets. Immunofluorescence and RNA analysis showed an increased insulin protein and mRNA levels in Sidt2^−/−^ mice. However, under stimulation with glucose, isolated islets from Sidt2 deficiency mice manifested significant decrease in insulin releasing than control. These data combined with significantly abundant insulin content remaining after high concentration of glucose and 30 mM KCL stimulation are sufficient to reveal that Sidt2−/− mice had an insulin secretion defect. Nevertheless, the increased insulin mRNA expression might also contribute to higher insulin content in KO mice.

Insulin granules were classified into four types based on their morphology [28]. These types include (a) mature granules with electron-dense core, (b) immature granules with electron-translucent cores, (c) granules with a crystal and, (d) empty granules, which presumably represent retrieval vesicles reminiscent of successful docking, fusion and release of their insulin cargo [26], [29], [30], [31]. The increasing number of mature granules, decreasing number of empty granules and rarity of docking and fusion of dense core granule with the plasma membrane also gave a proof of defective insulin exocytosis in Sidt2 deficiency mice at morphology. Hypertrophic volumes of islets, increased quantities of large size islets, and elevated numbers of insulin secretory granules may indicate compensation of islets to decreased insulin secretion.

Interestingly, the pathologic effects of in vivo Sidt2 deficiency are very similar to those of another lysosomal membrane protein, Synaptagmin-7, e.g. impaired insulin secretion, glucose intolerance [32], muscle fiber invasion by leukocytes and muscle weakness (our data unreported) [33]. Synaptagmin-7 acts as a Ca^2+^ sensor not only in the specialized glucose-induced insulin secretory granule release [32], but also in the ubiquitous process of Ca^2+^-regulated lysososmal exocytosis[33]. Notably, another lysosomal membrane protein, Mucolipin 1, the type IV mucolipidosis-associated protein, acts as an endolysosomal iron and calcium release channel[34], [35], whose deficiency also led to defective lysosomal exocytosis in skin fibroblast [36]. In consideration that Sidt2 is a multipass transmembrane protein and estimated to be a RNA transporter by gene ontology [18], it was reasonable to speculate that Sidt2 functions as another cation channel on lysosomal membrane. Nutrient-induced increases in intracellular free Ca^2+^ concentrations from multiple resource, both extracellular and intracellular, are the key trigger for insulin release from pancreatic islet beta-cells [37], [38]. In the β cells of Sidt2 KO mice, stimulation may result in a weaker increase of intracellular free Ca^2+^ because of loss of Ca^2+^ resource from lysosomes compared with normal mice, consequently deficiency of insulin secretion. More experiments on cell calcium imaging and patch-clamp of β cells from Sidt2 KO mice need to be carried to prove this hypothesis.

In summary, we showed that Sidt2 deficient mice have impaired insulin secretion and glucose tolerance. Sidt2 may be another novel cellular protein responsible for impaired glucose tolerance seen in human diabetes. This study furthers our understanding in the maintenance of glucose homeostasis.
